# Seniors' Physical Activity in Neighborhood Parks and Park Design Characteristics

**DOI:** 10.3389/fpubh.2020.00322

**Published:** 2020-07-29

**Authors:** Yujia Zhai, Dongying Li, De Wang, Cheng Shi

**Affiliations:** ^1^College of Architecture and Urban Planning, Tongji University, Shanghai, China; ^2^Department of Landscape Architecture and Urban Planning, College of Architecture, Texas A&M University, College Station, TX, United States

**Keywords:** neighborhood park, design characteristic, senior, walking, energy expenditure, pedometer

## Abstract

Physical activity brings multiple health benefits to seniors. Neighborhood parks provide seniors with accessible spaces and opportunities to engage in physical activity. This study investigated the associations between neighborhood park design characteristics and seniors' total walking step and energy expenditure during the park visit. Seniors' total step was measured by pedometer, and energy expenditure was calculated based on self-reported activities in the park. The study was conducted in 15 neighborhood parks with an area <10 ha, and included 234 senior participants. One-way ANOVA analyses indicated that seniors in parks with larger surface area, longer trail, larger natural area and outdoor fitness equipment had taken more steps. While seniors in parks without water expended more energy. For instance, seniors in parks with surface areas <3 ha walked 507 fewer steps than seniors in parks with areas between 3 and 5 ha, and 691 fewer steps than those in parks larger than 5 ha. When including seniors' demographic attributes, multiple regression analyses suggested that total step was negatively associated with age, but positively associated with total natural area in the park and the presence of outdoor fitness equipment. Seniors energy expenditure was positively associated with BMI and the presence of outdoor fitness equipment. Energy expenditure was also related to income. These findings provide direct implications for neighborhood park design and management. Planners and designers can include more natural areas over paved areas, create longer trails and place more outdoor fitness equipment in parks to encourage seniors to walk and spend more energy.

## Introduction

Physical inactivity increases the risk of various chronic diseases, e.g., diabetes, cerebrovascular diseases, and obesity, which represent leading causes of death in the senior population. Conversely, appropriate levels of activity provide multiple benefits to seniors' physical and mental health (1, 2). Despite these facts, it is challenging to encourage seniors to stay physically active. Providing a safe, barrier-free, and healthy built environment for activities is critical to encouraging seniors' physical activity.

Urban parks are outdoor environments that facilitate physical activity for all ages (3–6), and they are also where seniors usually choose to engage in physical activity (7). Seniors prefer natural environments than built environments more than other adults (8, 9). Seniors tend to be physically active during park visits, and spend half their time walking (10). Existing research indicates that seniors prefer neighborhood park without nuisance, with many trees and plants (11). However, the characteristics of seniors' physical activity in urban parks have not been thoroughly explored, and their needs in the urban park are not well understood (4, 12–14).

Existing research has examined the relationship between parks and physical activity from two directions. The first is how park characteristics relate to residents' overall physical activity at the neighborhood level, such as the amount of moderate and vigorous physical activity of residents in 1 week (3), and whether residents achieve recommended levels of activity (15). Important park characteristics that encourage physical activity at the neighborhood level are: more parks and green space (16), larger park size (6), more features in the park (17), and proximity of residents to parks (3, 4). These findings support the inclusion of more green space and park facilities in community planning and policy-making. The second perspective is how different park activity zones encourage moderate and vigorous physical activity at the activity zone level, such as the numbers of individuals engaging in moderate and vigorous activities on park pathways or in open spaces (18). Trails have the strongest relationship with park use for walking and other physical activities (17, 19, 20). However, considering activity at the zone level does not allow researchers to capture an individual's total physical activity in the park. It is important to understand how park characteristics may influence physical activity at the level of individual visitors. Without this information, urban designers and green space managers lack key guidance on how to design a neighborhood park to maximize its health benefits.

Another potential limitation of existing studies is the methods by which physical activity is captured. Widely-used methods include self-reported activity and on-site observation. The self-reported approach asks participants to record their physical activity during a period, such as whether they visited the park for physical activity and which specific park facilities they used (17), and the duration of physical activity (21–23). However, these methods are susceptible to recall bias and may not accurately represent actual activity levels (5, 24). On-site observation tools, such as the System for Observing Play and Recreation in Communities (SOPARC), can be used to examine differences in physical activity level between various park activity zones (25, 26). Using this tool, researchers scan the whole activity zone at sampling moments and count the numbers of visitors engaging in physical activities with different intensities. However, scanning may not be effective in heavily-used urban parks full of people, and accurate recording is often costly and time-consuming (5). Therefore, objective approaches that can efficiently collect data on park users' physical activity are needed (5, 21). Equipment such as pedometers and accelerometers have been used to measure the intensity of physical activity (5, 27, 28), but few studies have employed them to examine physical activity in urban parks.

Furthermore, few studies have addressed how park characteristics may impact physical activity from a design perspective (29). Designers are interested in knowing how many paved open spaces should be provided, how the trails should be distributed, and how large natural areas such as lawns and groves should be. However, existing findings may only suggest the presence of trail that would facilitate physical activity, rather than addressing design characteristics of these features, thus may have limited implications for park design. Therefore studies that can bear design implications and inform design practices are needed (5, 26, 30, 31).

This study aims to address the above-mentioned knowledge gaps by examining how neighborhood park design characteristics relate to seniors' walking and energy expenditure on park visit at the individual level. We used pedometers to measure seniors' total steps taken during their park visits and estimated energy expenditure based on their recall of the activities they engaged in. This study can provide empirical evidence on how neighborhood park design attributes may relate to the physical activity of seniors in parks as well as providing an approach for collecting physical activity data. Moreover, the research findings can inform future urban park design and management to promote physical activity in seniors.

## Methods

### Study Sites

**Figure 1:**
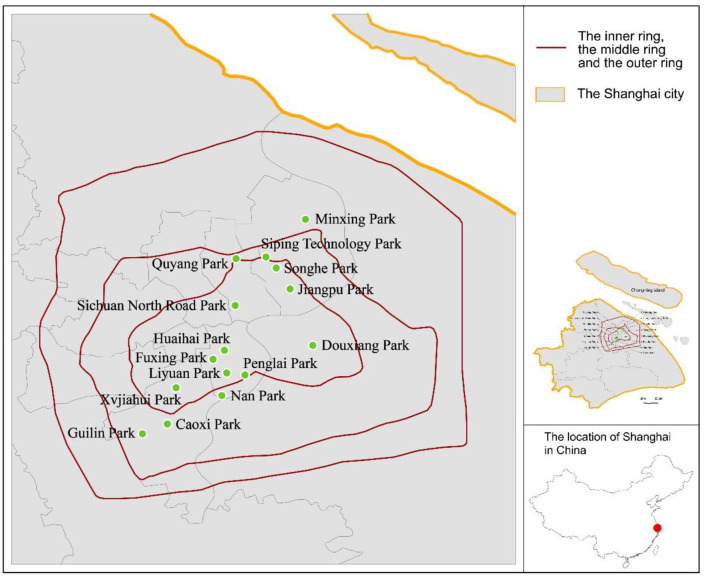
Locations of the 15 neighborhood parks in Shanghai.

**Figure 2:**
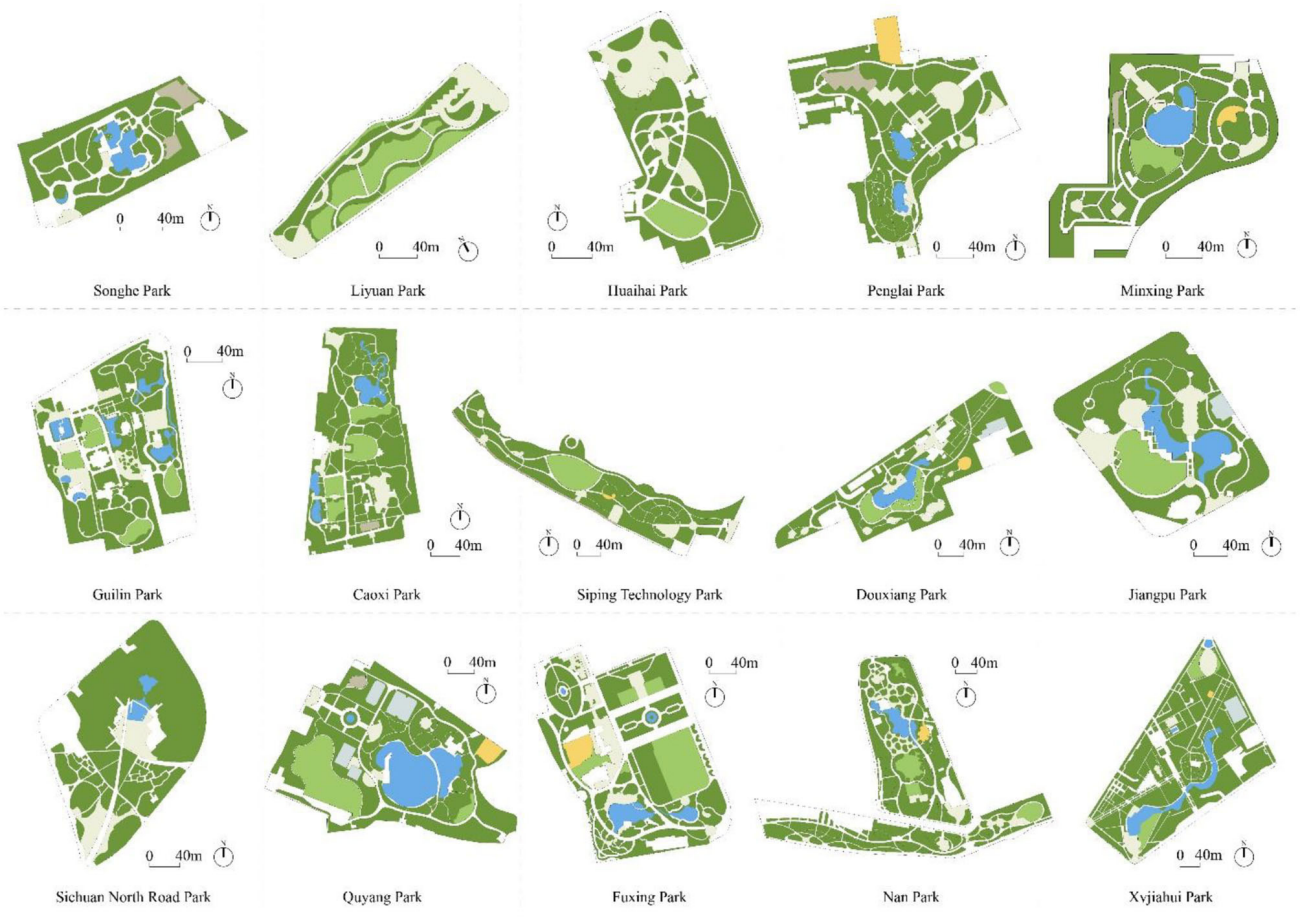
Master Plans of the 15 neighborhood parks. [Figure credit: authors; an adapted version of figure has been published in article that belong to the same project (13).]

Neighborhood parks provide seniors with accessible outdoor spaces to engage in physical activities. Fifteen neighborhood parks in the city of Shanghai were selected as study sites (Table 1, Figure 1). Shanghai is the second-largest city in China, with an area of ~6,300 km^2^ and a population of 24 million people at the end of 2016 (33). Its population density is very high, with 18,000 to 32,000 residents per km^2^ in the central districts (33). Three main ring-shaped roads (the inner ring, the middle ring and the outer ring) divide Shanghai into four parts, and the 6th National Census of Population shows that population density decreases from the area within the inner ring to the area beyond the outer ring (34). The city has 165 urban parks with a total area of 24 km^2^ (32). Due to limited land availability, most urban parks in Shanghai have a small surface area (35); around half of them are <5 ha (36). Fifteen neighborhood parks with surface areas of between 3 ha and 10 ha were selected as study sites based on the following criteria: 1. the park administrators approved data collection; 2. detailed digital survey documents of the park were obtainable; and 3. the park served the general public and was open to all. The 15 neighborhood parks all have common activity zones, such as lawns, trails, and paved open spaces (Figure 2). All parks are either within or very close to the middle ring and are frequently used by the citizens. Annual visitor numbers from 2015 for the selected parks ranged from 186,016 to 12,157,350.

**Table 1:** Selected urban parks.

**Area category**	**No**.	**Park name**	**Area (ha)**	**Number of visitors in 2015 (32)**	**District**
Park area <3 ha	1	Songhe Park	1.6	712,604	Yangpu district
	2	Liyuan Park	1.7	323,092	Huangpu district
	3	Huaihai Park	2.5	1,942,450	Huangpu district
3 ha ≤ Park area <5 ha	4	Penglai Park	3.2	186,016	Huangpu district
	5	Minxing Park	3.2	834,982	Yangpu district
	6	Guilin Park	3.6	242,040	Xvhui district
	7	Caoxi Park	3.8	624,430	Xvhui district
	8	Siping Technology Park	3.8	398,122	Yangpu district
	9	Douxiang Park	3.8	291,363	Pudong new district
	10	Jiangpu Park	3.8	1,228,734	Yangpu district
	11	Sichuan North Road Park	4.5	10,723,216	Hongko district
5 ha ≤ Park area <10 ha	12	Quyang Park	6.2	1,468,108	Hongko district
	13	Fuxing Park	6.5	7,515,059	Huangpu district
	14	Nan Park	8.6	1,012,700	Huangpu district
	15	Xvjiahui Park	8.9	12,157,350	Xvhui district

### Procedure

The study was conducted on sunny or cloudy weekdays during 3 weeks in October of 2017, when the weather in Shanghai is conducive to outdoor activities. Sampling days consisted of two data collection sessions, one in the morning (9:00 am−12:00 pm) and one in the afternoon (1:00 pm−5:00 pm). Researchers were stationed at the most frequently-used park entrance and invited seniors entering the park to voluntarily participate in the study. Three criteria were used in screening participants: 1. The participant should be aged 60 and above, 2. The participant did not need walking aids, and 3. The participant planned to visit the park, rather than pass through. Once a senior park user agreed to participate in the study, he or she was asked to sign a consent form and provided with the researcher's telephone number. The participant's telephone number was also recorded with their approval.

When distributing pedometers, the researcher turned on the pedometer and made sure it had been reset, and helped the participant put it around their neck or on their waist, where pedometer has a high reliability (37). We also instructed the participant not to touch the buttons on pedometers to prevent them from turning it off or resetting the record by accident. When pedometers were returned, the researcher recorded the total steps measured, as well as the time of return in order to discriminate step data from different participants. At the time of return, participants were invited to complete a questionnaire addressing their demographic information, daily park use patterns, and physical activities in the park. We asked participants to recall and report in chronological order each activity they engaged in and the duration of each activity.

### Measures

#### Total Steps and Energy Expenditure in Parks

The physical activity of senior park users was assessed using the total steps measured by pedometer and by self-reported energy expenditure. The pedometer used in this study was the Yamax Power Walker EX-510 (Yamax Corp., Tokyo, Japan), which showed a high accuracy in counting steps (37). Existing research has proven the validity of pedometer in measuring physical activity (27, 28). In particular, the Yamax pedometer has been shown to be very accurate in recording steps and distance (38), and in counting the steps of seniors who neither uses walking aids nor walk very slow (39). Specifically, when walking speed is >0.83 m/s, its step counts have acceptable error rates (40). Typically, seniors aged 60 and above have a walking speed >1 m/s (41), thus it is appropriate to measure their steps with a pedometer.

Energy expenditure was calculated based on participants' reported activity types and durations. Using the Compendium for Physical Activities, we identified the metabolic equivalent (MET) intensity level for each type of activity reported by participants. The MET is a standardized measure of activity intensity defined as the ratio of work to resting metabolic rate. For example, walking is estimated as 3 METs and running is considered to be 6 METs. We calculated the energy expenditure of each participant for each activity by multiplying their weight (kg), the energy cost (METs) of a given physical activity (kcal·kg^−1^·h^−1^), and the duration of the physical activity (h).

#### Park Design Characteristics

**Figure 3:**
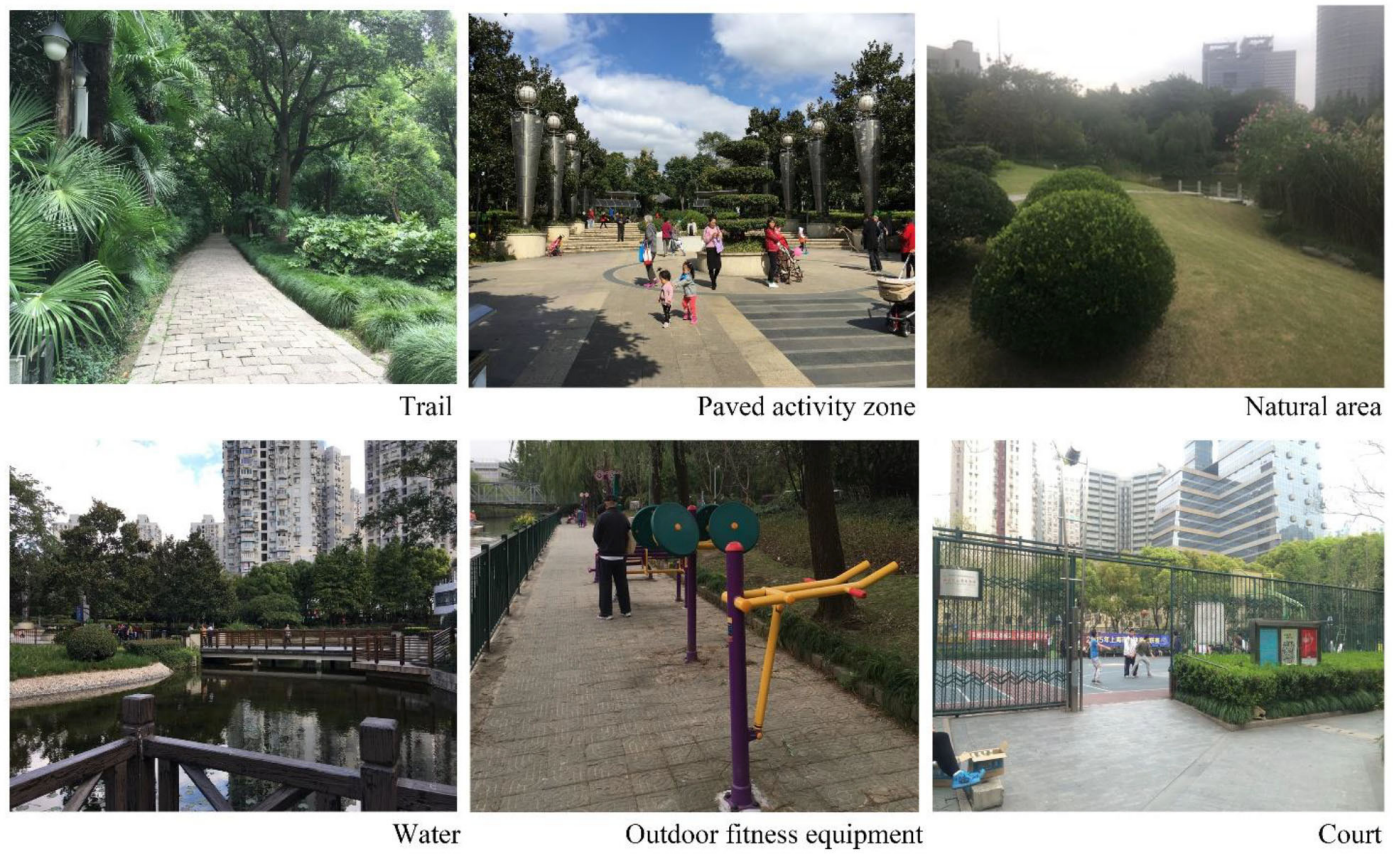
Park scenes of the study parks.

**Figure 4:**
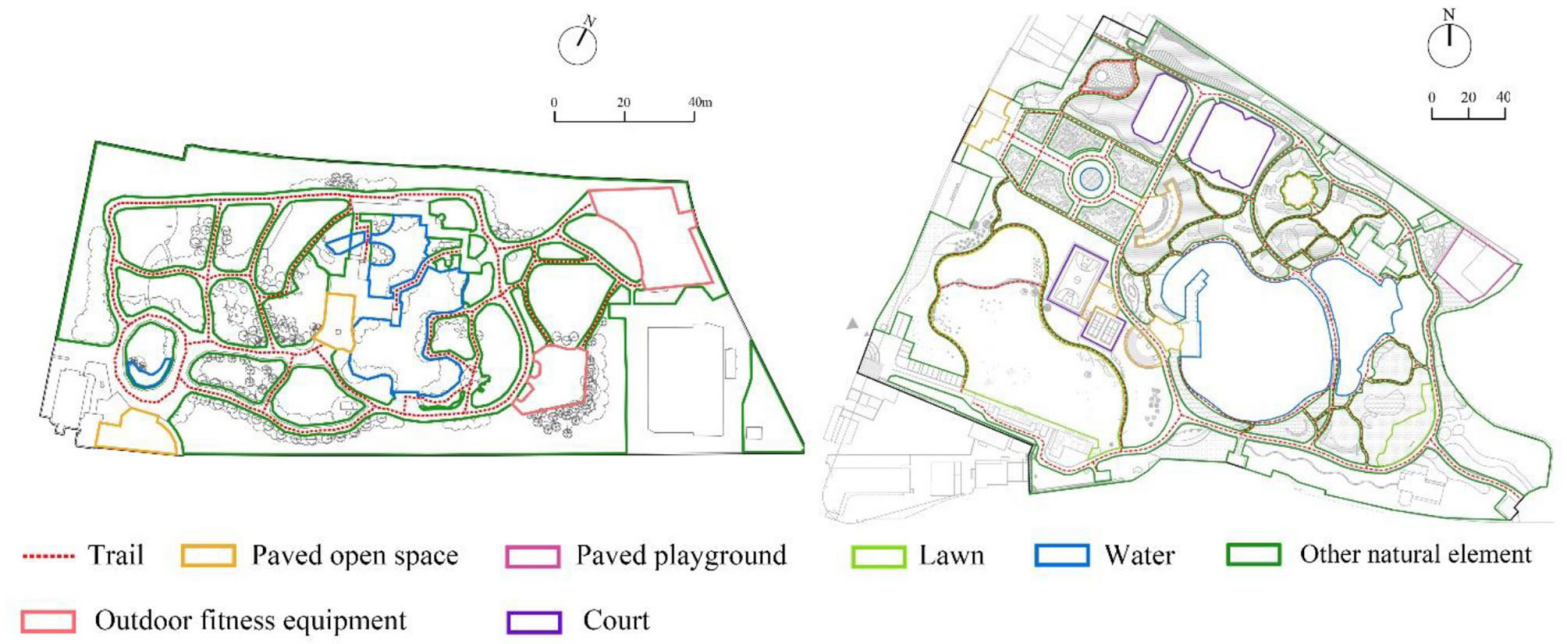
Park activity zone type identification of Songhe Park (left) and Quyang Park (right).

We measured seven park design characteristics: 1. Park area, 2. Total trail length, 3. Total paved activity zone area, 4. Total natural area, 5. Presence of water, 6. Presence of outdoor fitness equipment, and 7. Presence of court (Figure 3). Table 2 provides the definition, justification, type, and data source for these design characteristics. We hypothesized that parks with larger areas, more trails, larger paved activity zone area, larger natural area, water features, outdoor fitness equipment, and courts would be associated with more steps and more energy expenditure for senior users. All design variables were measured based on surveys of parks in AutoCAD format provided by the Shanghai Greening Administration Bureau and local park administrators. The researchers visited all 15 parks, field-validated the survey drawings, corrected errors, identified activity zone types, and then calculated values for each park's design characteristic variables using AutoCAD (Figure 4).

**Table 2:** Seniors' physical activity variables and neighborhood park design variables.

		**Definition/measurement**	**Justification**	**Variable type**	**Data source**
**Seniors' physical activity variables**					
	1. Total step	Total steps seniors walked during the park visit	–	Continuous	Pedometer
	2. Energy expenditure	Total energy senior expended during the park visit	–	Continuous	Questionnaire/calculated based on compendium of Physical Activities
**Neighborhood park design variables**					
	1. Park area	Surface area of the entire park	Large parks tend to have more features (42), which may encourage more physical activities.	Categorical (<3 ha, 3–5 ha and ≥5 ha)/Continuous	Park AutoCAD map/site visit
	2. Total trail length	Total length of all trails in the park	Parks with a track appeared to draw more seniors (4).	Categorical (<1 km, 1–2 km and ≥2 km)/Continuous	Park AutoCAD map /site visit
	3. Total paved activity zone area	The total area of all paved activity zones in the park, e.g., open space, court and paved children playground.	Larger activity zone appeared to attract more users (43).	Categorical (<0.4 ha, 0.4–0.6 ha and, ≥0.6 ha)/Continuous	Park AutoCAD map /site visit
	4. Total natural area	Area of natural elements, e.g., water, lawn, grove.	Nature experience could benefit mental health (44, 45). Adolescents exposed to more nature have a better daily mood (46).	Categorical (<2 ha, 2–4 ha and, ≥4 ha)/Continuous	Park AutoCAD map /site visit
	5. Presence of water	Presence of water in the park	Water contributes to a better mood (47, 48).	Categorical (0 = without, 1 = with)	Park AutoCAD map /site visit
	6. Presence of outdoor fitness equipment	Presence of fitness equipment in the park	Outdoor fitness equipment attracts a lot of senior users (49, 50) and contributes to the increase of moderate and vigorous physical activity (51).	Categorical (0 = without, 1 = with)	Park AutoCAD map /site visit
	7. Presence of Court	Presence of court in the park	Use of courts facilitates physical activity in the park (4, 18, 52)	Categorical (0 = without, 1 = with)	Park AutoCAD map /site visit

### Statistical Analyses

Statistical Analyses were conducted using IBM SPSS Statistics. Descriptive statistics were used to determine general characteristics of the collected sample data and park design characteristics. One-way ANOVA tests were then fitted to test whether seniors' physical activity, including total steps and energy expenditure, differed between parks with different design characteristics. In ANOVA analysis, park design characteristics were coded as categorical variables (Table 2). Park area was classified as smaller than 3 ha, 3–5 ha, and larger than 5 ha; trail length as <1 km, 1–2 km, and longer than 2 km; paved activity zone areas as <0.4 ha, 0.4–0.6 ha, and larger than 0.6 ha; and natural area as <2 ha, 2–4 ha, and larger than 4 ha.

We then used regression models to examine the relationships between park design characteristics and seniors' walking step and energy expenditure. In the analyses, variables of park area, total trail length, total paved activity zone area and total natural area were coded as continuous variables, other park design variables, including presence of water, presence of outdoor fitness equipment, and presence of court were coded as categorical variables. First, we examined whether our data demonstrated a multi-level structure (i.e., park participants nested within parks). If so, mixed models would be required for analysis. However, when we calculated the intra-class correlation coefficient, we found the between-cluster variance to be very small (ICC = 0.050 for total steps and ICC = 0.038 for energy expenditure); this ruled out any need for a mixed model. We fitted linear regression models to predict total steps and energy expenditure using park design characteristics and included seniors' demographic attributes as control variables. Total steps and energy expenditure were log-transformed, as they displayed right-skewed distributions. Since park design characteristic variables exhibited collinearity, stepwise models selection were applied for the both models.

## Results

### Descriptive Statistics

Total of 257 senior park users participated in the study (Table 3). Those who returned with the pedometer turned off, or whose survey results were inconsistent with activity durations recorded on the pedometer, were excluded from the study. A total of 234 (91.05%) participants had valid pedometer data and demographic information and were included in the analysis. On average, each park had 16 valid senior participants (*Min* = 11, *Max* = 23, *SD* = 3.54). As indicated in Table 3, the average age of participants was around 70 years old (*Min* = 60, *Max* = 93, *SD* = 7.54) and the average BMI of participants was 23.45 (*Min* = 12.37, *Max* = 31.25, *SD* = 2.89). One hundred and thirty-two (56.4%) participants were male, 188 (80.3%) lived with their spouse, and 112 (47.9%) had a household monthly income between 5,000 and 10,000 CNY (749-1498 USD). Eighty-six (36.8%) seniors reported that their health was excellent or good, and 132 (56.4%) seniors felt their health was fair. One hundred and sixty-seven (71.4%) participants claimed that they came to the park for exercise, and 51 (21.8%) said they came with multiple purposes.

**Table 3:** Descriptive statistics for senior participants.

	**Minimum**	**Maximum**	**Mean**	**Std. deviation**
1. Age	60.00	93.00	69.52	7.540
2. Height (m)	1.48	1.84	1.64	0.075
3. Weight (km)	30.50	90.00	63.54	9.859
4. BMI	12.37	31.25	23.45	2.894
		**Frequency**	**Percent**	
5.Gender	Male	132	56.4	
	Female	102	43.6	
6. Marital status	Single	46	19.7	
	Not single	188	80.3	
7. Houshold monthly income (CNY)	<3,000	31	13.2	
	3,000–5,000	65	27.8	
	5,000–10,000	112	47.9	
	10,000–20,000	26	11.1	
8. Self-reported health condition	Excellent	38	16.2	
	Good	48	20.5	
	Fair	132	56.4	
	Bad	16	6.8	
9.Park visit purpose	Exercise	167	71.4	
	Contact with nature	14	6.0	
	Meet friends	2	0.9	
	Multiple	51	21.8	

Out of all 234 participants with valid data, 191 recalled the types of physical activity they engaged in and the duration of each type of physical activity. On average, each participant walked 2,278 steps in the park (*Min* = 158, *Max* = 10,320, *SD* = 1,642.403). Of these 191 participants, one (0.52%) engaged in four types of physical activities; 11 (5.76%) engaged in three kinds of physical activities; 82 (42.93%) took parts in two kinds of physical activities; and 97 (50.79%) participated in only one type of physical activity. In total, participants reported 27 types of physical activities, the most frequently mentioned of which were walking (139 seniors, 72.77%), meeting and chatting with friends (29 seniors, 15.18%), and using outdoor fitness equipment (20 seniors, 10.47%). We calculated the energy expenditure of each participant for each activity by multiplying their weight (kg), the energy cost of a given physical activity (kcal·kg^−1^·h^−1^), and the duration of the physical activity (h^−1^). The total energy expenditure of a participant was then calculated as the sum of energy expenditures across all kinds of activities they performed. On average, each senior expended 148.27 kcal (*Min* = 14.50, *Max* = 1007.40, *SD* = 113.579) during their stay in the park.

### Park Design Characteristics

Table 4 reports descriptive statistics for park design characteristics. On average, the 15 neighborhood parks had a surface area of 4.4 ha (*Min* = 1.61, *Max* = 8.92, *SD* = 2.219), a total trail length of 2.27 km (*Min* = 0.533, *Max* = 6.05, *SD* = 1.339), a total paved activity area of 0.49 ha (*Min* = 0.12, *Max* = 0.86, *SD* = 0.204), and a total natural area of 2.68 ha (*Min* = 0.96, *Max* = 5.00, *SD* = 1.218). Twelve parks (80%) had water features, six parks (40%) had outdoor fitness equipment, and four parks (26.7%) contained courts. Correlation analyses were used to detect associations between park design characteristics (Table 5). The results indicated that park area was positively associated with trail length, *r* (14) = 0.888, *p* < 0.001, total paved activity zone area, *r* (14) = 0.660, *p* < 0.005, and total natural area, *r* (14) = 0.962, *p* < 0.001. Parks with larger natural area also have longer trails *r* (14) = 0.858, *p* < 0.001, and larger total paved activity zone area, *r* (14) = 0.588, *p* < 0.005. Parks with courts also tend to have larger paved activity zone area, *r* (14) = 0.523, *p* < 0.005.

**Table 4:** Descriptive statistics for park design characteristics.

	***N***	**Minimum**	**Maximum**	**Mean**	**Std. deviation**
Park area (m^2^)	15	16058.52	89172.96	44045.893	22192.869
Total trail length (m)	15	533.27	6048.12	2268.229	1339.746
Total paved activity zone area (m^2^)	15	1176.88	8589.45	4925.184	2048.820
Total natural area (m^2^)	15	9613.77	50033.88	26764.212	12176.843
		**Frequency**	**Percent**		
Presence of water	No water	12	80.0		
	With water	3	20.0		
Presence of outdoor fitness equipment	No fitness equipment	6	40.0		
	With outdoor fitness equipment	9	60.0		
Presence of court	No court	4	26.7		
	With court	11	73.3		

**Table 5:** Correlation matrix for park design characteristic variables.

	**Park area**	**Total trail length**	**Total paved activity zone area**	**Total natural area**	**Presence of water**	**Presence of outdoor fitness equipment**
Total trail length^p^	0.888^**^					
Total paved activity zone area^p^	0.660^**^	0.454				
Total natural area^p^	0.962^**^	0.858^**^	0.588^**^			
Presence of water^S^	0.424	0.463	0.116	0.386		
Presence of outdoor fitness equipment^S^	−0.346	−0.157	−0.409	−0.157	0.068	
Presence of Court^S^	0.489	0.209	0.523^**^	0.384	0.302	−0.185

p *Pearson correlation coefficients*;

S *Spearman correlation coefficients*.

** *p < 0.05 (2-tailed)*.

### Does Seniors' Total Step and Energy Expenditure Differ in Parks With Different Design Characteristics?

**Figure 5:**
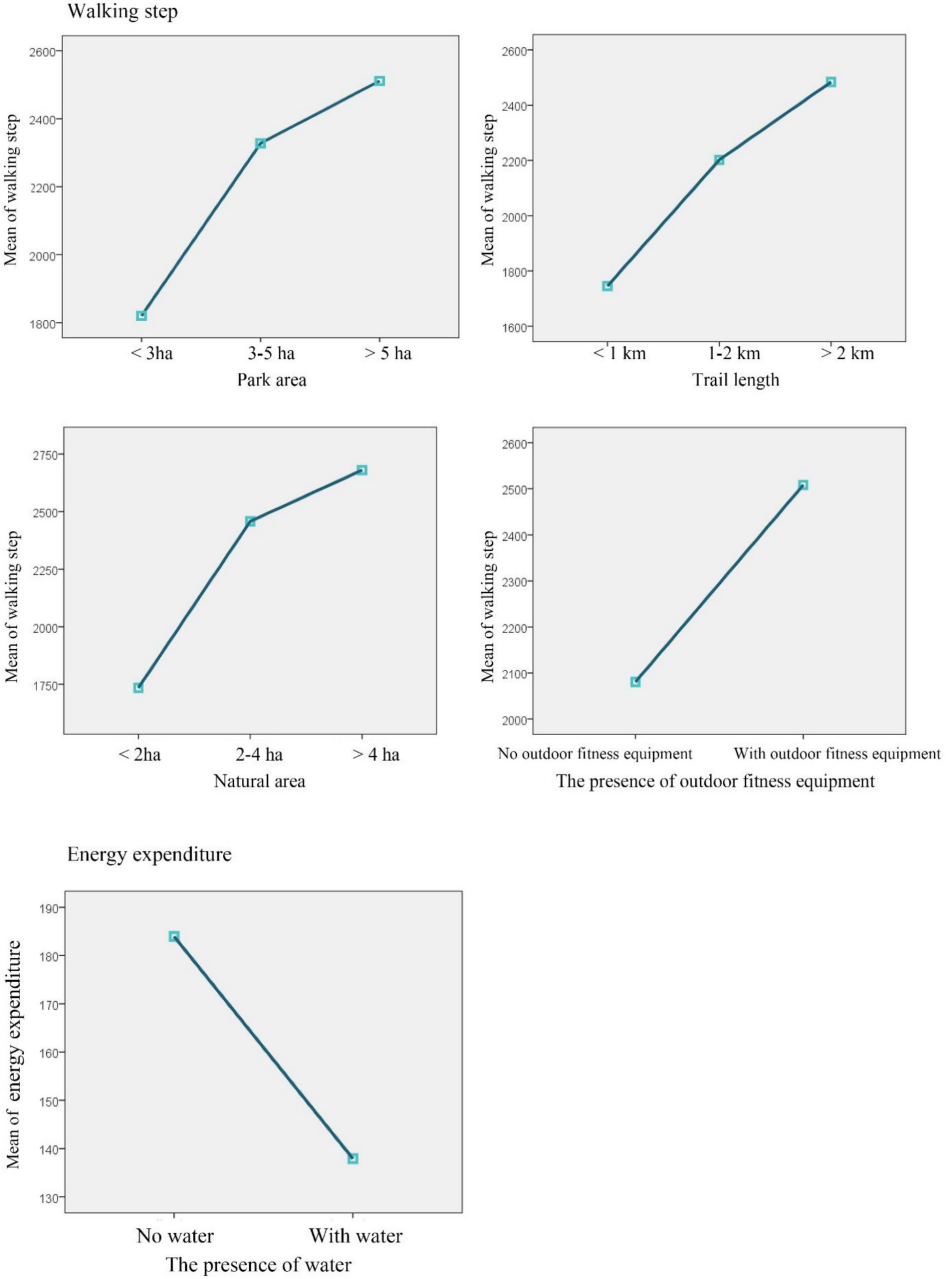
Differences in seniors' total walking step and energy expenditure in parks with different design characteristics.

ANOVA analyses were performed to detect the differences in seniors' mean total step and mean energy expenditure in parks with different design characteristics. The results indicated that on average, seniors walk more steps in parks with larger surface area, *F*_(2, 231)_ = 2.45, *p* = 0.089, longer trail, *F*_(2, 231)_ = 2.85, *p* = 0.060, larger natural area, *F*_(2, 231)_ = 6.27, *p* = 0.002, and outdoor fitness equipment, *F*_(1, 231)_ = 4.00, *p* = 0.047 (Table 6, Figure 5). In particular, ANOVA *post hoc* (LSD) analyses indicated that senior participants in parks with <3 ha total area walked 507 fewer steps than those in parks with areas between 3 and 5 ha (*p* = 0.074), and 691 fewer steps compared to seniors in parks larger than 5 ha (*p* = 0.032) (Table 7). On average, seniors in parks with more than 2 km of trails walked 739 more steps (*p* = 0.021) than seniors in parks with <1 km of trails. Similarly, seniors in parks with <2 ha of natural area walked 724 fewer steps than those in parks with between 2 and 4 ha of natural area (*p* = 0.003), and 946 fewer steps (*p* = 0.002) than those in parks with more than 4 ha of natural area. Seniors expended more energy in parks without water, *F*_(1, 189)_ = 5.608, *p* = 0.019, but no significant differences in seniors' average energy expenditure were detected between parks with different sizes*, F*_(2, 188)_ =.725, *p* = 0.486, trail lengths, *F*_(2, 188)_ = 1.101, *p* = 0.335, paved activity zone areas, *F*_(2, 188)_ = 0.755, *p* = 0.472, natural areas, *F*_(2, 188)_ = 0.188, *p* = 0.889, presence of outdoor fitness equipment, *F*_(1, 189)_ = 1.081, *p* = 0.300, or presence of court, *F*_(1, 189)_ = 0.014, *p* = 0.906.

**Table 6:** ANOVA analysis for seniors' total step, energy expenditure and park design characteristics.

		**Steps**					
		**Sum of squares**	**df**	**Mean square**	***F***	**Sig**.	**Sum of squares**
Park area	Between groups	13055887.570	2	6527943.785	2.450	0.089^*^	18761.540
	Within groups	615458633.300	231	2664323.088			2432275.131
	Total	628514520.900	233				2451036.671
Total trail length	Between groups	15140996.130	2	7570498.067	2.851	0.060^*^	28385.552
	Within groups	613373524.800	231	2655296.644			2422651.119
	Total	628514520.900	233				2451036.671
Total paved activity zone	Between groups	4417467.558	2	2208733.779	0.818	0.443	19517.993
	Within groups	624097053.300	231	2701718.846			2431518.678
	Total	628514520.900	233				2451036.671
Total natural area	Between groups	32350036.860	2	16175018.430	6.267	0.002^**^	3074.281
	Within groups	596164484.000	231	2580798.632			2447962.390
	Total	628514520.900	233				2451036.671
Presence of water	Between groups	30279.745	1	30279.745	0.011	0.916	70633.114
	Within groups	628484241.200	232	2708983.798			2380403.557
	Total	628514520.900	233				2451036.671
Presence of outdoor fitness equipment	Between groups	10642189.750	1	10642189.750	3.996	0.047^**^	13934.383
	Within groups	617872331.200	232	2663242.807			2437102.288
	Total	628514520.900	233				2451036.671
Presence of court	Between groups	3754170.004	1	3754170.004	1.394	0.239	181.134
	Within groups	624760350.900	232	2692932.547			2450855.537
	Total	628514520.900	233				2451036.671

* *p < 0.10 (2-tailed)*.

** *p < 0.05 (2-tailed)*.

**Table 7:** AOVA *post hoc* (LSD) analysis for total step and neighborhood park design characteristics.

	**(I)**	**(J)**	**Mean difference (I-J)**	**Std. error**	**Sig**.
Park area	<3ha	3–5 ha	−507.255	282.882	0.074^*^
		≥5 ha	−690.803	320.756	0.032^**^
	3–5 ha	≥5 ha	−183.548	253.954	0.471
Trail length	<1 km	1–2 km	−456.819	332.383	0.171
		≥2 km	−738.972	317.174	0.021^**^
	1–2 km	≥2 km	−282.154	234.274	0.230
Natural area	<2 ha	2–4 ha	−723.567	240.629	0.003^**^
		≥4 ha	−945.942	305.279	0.002^**^
	2–4 ha	≥4 ha	−222.374	281.796	0.431

* *p < 0.10 (2-tailed)*.

** *p < 0.05 (2-tailed)*.

### Do Relationships Between Seniors' Total Step and Energy Expenditure and Neighborhood Park Design Characteristics Still Hold When Controlling for Demographic Attributes?

The relationship of park design characteristics and seniors' total steps and energy expenditures were evaluated using multiple stepwise regression analyses (Table 8). We included age, gender, BMI, family income, health condition, and visit purpose as control variables. The multiple stepwise regression models indicated that seniors' walking step was negatively associated with senior age (β = −0.164, *p* = 0.011), but positively associated with natural area (β = 0.158, *p* = 0.015) and the presence of outdoor fitness equipment (β = 0.149, *p* = 0.021) in the park. These three factors explained 6.4% (*p* = 0.002) of the variance in step counts in the parks. Seniors' energy expenditure was positively related to BMI (β = 0.241, *p* = 0.001), household monthly income between 5,000 and 10,000 (β = 0.160, *p* = 0.024) and the presence of outdoor fitness equipment (β = 0.161, *p* = 0.024) in the park, these three factors explained 9.9% of the variance (*p* = 0.000) in senior energy expenditure.

**Table 8:** Total step, energy expenditure, and park design characteristics and senior demographic attributes (stepwise model).

	**Variables**	**Coef. (B)**	***SE***	***St. Coef. (β*)**	**Sig**.	**VIF**	**Overall model**
**Total step**							
	(Constant)	3.592	0.213		0.000		*R^2^* = 0.064 *Sig*. = 0.002
	Age	−0.008	0.003	−0.164	0.011	1.005	
	Total natural area	4.889E-6	0.000	0.158	0.015	1.017	
	Presence of outdoor fitness equipment	0.105	0.045	0.149	0.021	1.015	
**Energy expenditure**							
		1.465	0.162		0.000		
	BMI	0.023	0.007	0.241	0.001	1.038	
	Household monthly income between 5,000 and 10,000	0.089	0.039	0.160	0.024	1.009	*R^2^* = 0.099 *Sig*. = 0.000
	Presence of outdoor fitness equipment	0.090	0.040	0.161	0.024	1.035	

*Total step and energy expenditure are log-transformed*.

## Discussion

This study examined the relationships between neighborhood park design characteristics and seniors' total steps and energy expenditure in the park, using both objective and self-reported measures. We sampled 15 different parks to enhance the generalizability of the results.

### Seniors' Age, Household Monthly Income, and Physical Activity

The results indicated that younger seniors walked more in the park, and seniors with a household monthly income between 5,000 and 10,000 have larger energy expenditure than those with a monthly income <3,000 CNY. Existing research indicates that the young elderly tend to engage in more physical activity than the old elderly in daily life (53), and our findings suggest that this difference existed in park visits. Human skeletal muscle atrophies with age (54), and seniors in the 50–59 age have a better physical function than those in the 60–69 and 70–79 age groups (55). Therefore, young seniors are expected to walk more in the park. The needs of seniors in different decades and with different physical capabilities should be considered in urban park design and management. Seniors with higher incomes are more likely to participate in health promotion programs (56), report better health (57), have lower body mass index, and engage in more moderate and vigorous physical activity (58). That might explain our findings that compared to seniors with a household monthly income <3,000 CNY, seniors with a household monthly income between 5,000 and 10,000 CNY expend more energy in the park. On the other hand, urban park provides free settings for seniors with low income to engage in physical activity, efforts are needed to encourage those seniors to actively use urban parks.

### Park Size, Natural Area, and Seniors' Walking

Our findings suggest that seniors in larger neighborhood parks walk more. However, existing research with adults indicates that park size is not associated with the chance whether a park is used for physical activity (17), and features rather than park size is more important for park-based physical activity (4). One possible explanation is that previous studies used binary measures of physical activity, while our study measured walking steps and energy expenditure using continuous variables, which allows for quantitative comparisons between parks. More time spent in a park has been shown to correlate with higher levels of physical activity (59). Larger parks provide more area to explore and may encourage seniors to stay and walk for longer periods. We also found that larger natural area was associated with more walking steps. The health benefits of walking in natural areas have long been recognized (60). Nature is important to seniors (61), and can help seniors release stress (62). Compared to other adults, seniors have a stronger motivation to walk in natural environment (8). Larger natural area may contribute to a better mood and attracted seniors to stay longer and walk more. The findings suggest that we can provide larger natural area to encourage seniors' walking in the park.

### Trail Length and Seniors Walking

We found total trail length is related to seniors' total steps in the park. Existing research suggests that parks with tracks draw more seniors (4). A study in Missouri finds that users of a trail longer than 0.25 mile are more likely to report an increase in physical activity (63). Trail in the park is where seniors walking on, and longer trail can provide more opportunities for seniors to walk thus may facilitate seniors walking. In parks of limited area, designers can distribute longer trails to facilitate senior walking; further investigation is needed to explore how the characteristics of park trails may facilitate walking by seniors (64).

### Outdoor Fitness Equipment and Seniors' Physical Activity

We found that the presence of outdoor fitness equipment was associated with total steps and energy expenditure of seniors. Fitness equipment can accommodate a variety of fitness goals, and their presence provides opportunities for physical activity beyond walking. Existing studies reported that outdoor fitness equipment in parks attracts senior users (49, 50), and seniors use this equipment with the primary motivation of exercising and improving health (7). Compared to other adults, seniors have less access to walking trails and indoor gyms (63). In contrast, outdoor fitness equipment can provide seniors with important exercise opportunities (50). Landscape architects can provide outdoor fitness equipment in urban parks to facilitate the physical activity of senior visitors, and how seniors use outdoor fitness equipment should be further explored.

### Limitations

The present study has some limitations that should be considered. The parks included in the study were neighborhood parks with areas of <10 ha, and thus the results may not be applicable to larger parks such as city parks, regional parks or natural reserves. Although we included 15 parks to ensure the representativeness of the sites, all are located in Shanghai, China. The patterns of park features and park use may differ in less-dense urban areas, in suburban and rural locations, and in other cultures. Additionally, we used a convenient sample of seniors visiting the parks who volunteered to participate in the study. It is uncertain whether this population group displayed characteristics that may influence the physical activity data systematically. Third, only step counts were measured, and only pedometers were used for measurement. Future studies should consider combining pedometers, accelerometers, heart-rate monitors, armbands, or multi-sensor devices for more accurate estimates of activity intensity, activity duration, and energy expenditure.

## Conclusion

This study aimed to investigate the associations between neighborhood park design characteristics and seniors' walking and energy expenditure during park visits. The results indicated that park area, total trail length, and total natural area of the park were positive predictors of more walking. The presence of outdoor fitness equipment also contributed to more walking and energy expenditure. In addition, demographic and socioeconomic factors, as well as BMI are related to seniors' activities in parks. For example, senior's total step count was negatively associated with age; and their energy expenditure was positively related to BMI. More energy was also expended by seniors with a monthly household income between 5,000 and 10,000 CNY as compared to those with a monthly household income of <3,000 CNY. These findings can be used to guide park design and management to promote walking and active recreation in parks. For instance, planners and designers can include more natural areas and less impervious areas, create longer trails, and provide more outdoor fitness equipment in parks, especially in parks that are located in communities that demonstrate greater inactivity and obesity. Park use patterns and the needs of seniors with diverse demographic attributes should also be considered and addressed in future park design and management practice.

## Data Availability Statement

The raw data can not be shared because sensitive location information is asked to be reserved by IRB.

## Ethics Statement

Our study has been approved by the Institutional Review Board of Tongji University and we confirm that the patients/participants provided their written informed consent to participate in this study.

## Author Contributions

All authors listed have made a substantial, direct and intellectual contribution to the work, and approved it for publication.

## Conflict of Interest

The authors declare that the research was conducted in the absence of any commercial or financial relationships that could be construed as a potential conflict of interest.
